# A Highly Effective Biomass-Derived Solid Acid Catalyst for Biodiesel Synthesis Through Esterification

**DOI:** 10.3389/fchem.2022.882235

**Published:** 2022-03-16

**Authors:** Songdang Zhang, Hu Pan, Jinshu Huang, Yuncong Li, Heng Zhang

**Affiliations:** ^1^ State Key Laboratory Breeding Base of Green Pesticide and Agricultural Bioengineering, Key Laboratory of Green Pesticide and Agricultural Bioengineering, State-Local Joint Laboratory for Comprehensive Utilization of Biomass, Center for Research and Development of Fine Chemicals, Ministry of Education, Guizhou University, Guiyang, China; ^2^ College of Biological, Chemical Science and Engineering, Jiaxing University, Jiaxing, China

**Keywords:** biodiesel, renewable bio-based catalyst, response surface methodology, sulfonation, esterification

## Abstract

Efficient valorization of renewable liquid biomass for biodiesel production using the desirable biomass-based catalysts is being deemed to be an environmentally friendly process. Herein, a highly active biomass-based solid acid catalyst (SiO_2_@Cs-SO_3_H) with renewable chitosan as raw material through sulfonation procedure under the relatively mild condition was successfully manufactured. The SiO_2_@Cs-SO_3_H catalyst was systematically characterized, especially with a large specific surface area (21.82 m^2^/g) and acidity (3.47 mmol/g). The catalytic activity of SiO_2_@Cs-SO_3_H was evaluated by esterification of oleic acid (OA) and methanol for biodiesel production. The best biodiesel yield was acquired by Response Surface Methodology (RSM). The optimized reaction conditions were temperature of 92°C, time of 4.1 h, catalyst dosage of 6.8 wt%, and methanol to OA molar ratio of 31.4, respectively. In this case, the optimal experimental biodiesel yield was found to be 98.2%, which was close to that of the predicted value of 98.4%, indicating the good reliability of RSM employed in this study. Furthermore, SiO_2_@Cs-SO_3_H also exhibited good reusability in terms of five consecutive recycles with 87.0% biodiesel yield. As such, SiO_2_@Cs-SO_3_H can be considered and used as a bio-based sustainable catalyst of high-efficiency for biodiesel production.

## 1 Introduction

In today’s society, due to the rapid development of industry, the accompanying sharp increase in the consumption of fossil fuels has led to a series of social problems including energy shortages and environmental pollution (Li et al., 2017; Li et al., 2019; Zhao et al., 2019; Hu et al., 2021). Therefore, it is of great significance to develop the renewable bioenergy of huge potential in terms of green and efficiency, aiming to replace the increasingly scarce traditional fossil energy (Liu et al., 2021; Li et al., 2022). Biodiesel, a kind of green and clean energy with low pollutant emission, non-toxic, good renewability and biodegradability, is regarded as a promising alternative to conventional fossil fuels (Atadashi et al., 2013; Pan et al., 2019; Tan et al., 2021). Biodiesel is mainly composed of various fatty acid alkyl esters, which are related to the composition of raw materials (Pan et al., 2018; Wang et al., 2018; Alagumalai et al., 2021). In general, biodiesel can be produced mainly through the transesterification of triglycerides of edible oil, vegetable oil, and waste edible oil, such as soybean (Hu et al., 2020; Xie et al., 2021b; Zhu et al., 2021), rapeseed (Hájek et al., 2020; Gaidukevič et al., 2021), and *Jatropha curcas* oil (Gutiérrez-López et al., 2021; Laskar et al., 2021), as well as the esterification of free fatty acids (FFAs) such as oleic acid (OA) with methanol. Recently, on account of the merits of microalgae, biodiesel production directly from microalgae oil has also attracted attention (Zhang et al., 2015; Bauer et al., 2017; Abdala et al., 2020). However, considering the issues of cost, environmental pollution, and food safety, producing biodiesel from waste edible oil has gradually become a research hotspot. It is worth noting that although the use of waste edible oil can reduce the relevant process cost and environmental pollution, the high levels of FFA in waste edible oil make its biodiesel technology difficult (Zhang et al., 2019; Bhatia et al., 2020; Shatesh et al., 2020). Therefore, to solve this problem, it is necessary to design a catalyst that can efficiently transform the high levels of FFA feedstock into biodiesel. Usually, the conventional catalysts used widely for biodiesel production are divided into two types, namely homogeneous and heterogeneous groups. Homogeneous catalysts restrict their further development due to the obvious disadvantages including difficult separation, difficult regeneration, and high requirements on experimental equipment (Guldhe et al., 2017; Negm et al., 2019; Li et al., 2020). Hence, heterogeneous catalysts are being regarded as the more extensive way for biodiesel production using low-quality oil feedstocks.

A negative saponification reaction occurs when biodiesel is produced directly from oil with high FFA content under the catalysis of an alkali catalyst. As such, acid pretreatment *via* esterification should be required to reduce acid content, and esterification of FFAs is also deemed as an effective manner to produce biodiesel (Zhang et al., 2017; Xie et al., 2021). To this end, it is a good choice to employ heterogeneous acid catalysts that can effectively catalyze the esterification reactions for the preparation of biodiesel. Solid acid catalysts containing sulfonic acid groups generally showed better catalytic activity for biodiesel production, especially in the esterification of FFAs with methanol. This should be attributed to the strong acidity of -SO_3_H (Liu et al., 2020). Nonetheless, compared with homogeneous catalysts, the heterogeneous acid catalysts usually depict lower catalytic efficiency, of which more stringent reaction conditions are required and the relatively higher catalyst production cost should also be carried out to achieve the desired effect. As a consequence, it is of great importance to developing low-cost and efficient solid acid catalysts that catalyze the esterification of FFAs to produce biodiesel.

With respect to this, biomass-derived catalysts have been widely used in the biodiesel production on account of their advantages of renewability, non-toxic, and environmental protection property. Nevertheless, the common biomass-based catalysts [bagasse (Akinfalabi et al., 2020), rice husk carbon (Chen et al., 2013) and bamboo (Tang and Niu, 2019), etc.] are mainly prepared by carbonization at high temperatures (>300°C) and sulfonation with corrosive sulfuric acid. The as-mentioned protocol usually requires harsh reaction conditions and also brings about pollution to the environment. Consequently, the relatively simple synthesis methods and the more environmentally friendly organic sulfonation reagents should be adopted to manufacture biomass-based catalysts. *p*-Toluenesulfonic acid, a non-oxidizing strong organic acid (one million times more acidic than benzoic acid), is considered to be a better organic sulfonation reagent (Wang et al., 2015).

Chitosan (Cs), the product of natural polysaccharide chitin after the removal of acetyl group, has demonstrated various attractive advantages such as biodegradability, biocompatibility, and non-toxicity. It is widely applied in many fields such as food additives, environmental protection, cosmetics, antimicrobial agent, drug development, and other daily chemical industry (Liu et al., 2015; Dhakshinamoorthy et al., 2021). Cs, as a weakly alkaline polysaccharide, is rich in -NH_2_ and -OH functional groups and also shows strong modification potential. Therefore, of particular interest is the rational synthesis of biomass-based catalysts from Cs for biodiesel production. Recently, supported solid acid catalysts have shown great application potential in the production of biodiesel owing to their excellent catalytic performance and strong design ability. With regard to this, nano-SiO_2_ is considered an excellent carrier because of its large pore size and specific surface area, along with strong stability even in the acidic media.

In this study, a novel biomass-derived solid acid catalytic material SiO_2_@Cs-SO_3_H was successfully prepared using *p*-toluenesulfonic acid as the sulfonation reagent, and nano-SiO_2_ as the solid support. The synthetic procedure used in this study was simple, mild, and environmentally friendly. Furthermore, the catalytic performance of SiO_2_@Cs-SO_3_H was assessed through the esterification of OA and methanol for biodiesel production.

## 2 Materials and Methods

### 2.1 Materials

Chitosan (>99%, the degree of deacetylation ≥95%), *p*-toluenesulfonic acid monohydrate (AR, 99%), and methanol (AR, ≥99.7%) were obtained from Chengdu Jinshan Chemical Reagent Co., Ltd. Oleic acid (AR, ≥99%) and nano-SiO_2_ were bought from Shanghai Macklin Biochemical Co., Ltd. Acetic acid (AR, ≥99.5%) was supplied by Chongqing Chuandong Chemical Co., Ltd. 50% of glutaraldehyde (AR, ≥99%) was purchased from Tianjin Damao Chemical Reagent Factory. Ethanol (AR, ≥99.5%) and petroleum ether (AR, 60–90°C) were obtained from Tianjin Fuyu Fine Chemical Co., Ltd. KOH (AR, ≥85.0%) was purchased from Shanghai Titan Scientific Co., Ltd.

### 2.2 Catalyst Preparation

**SCHEME 1:**
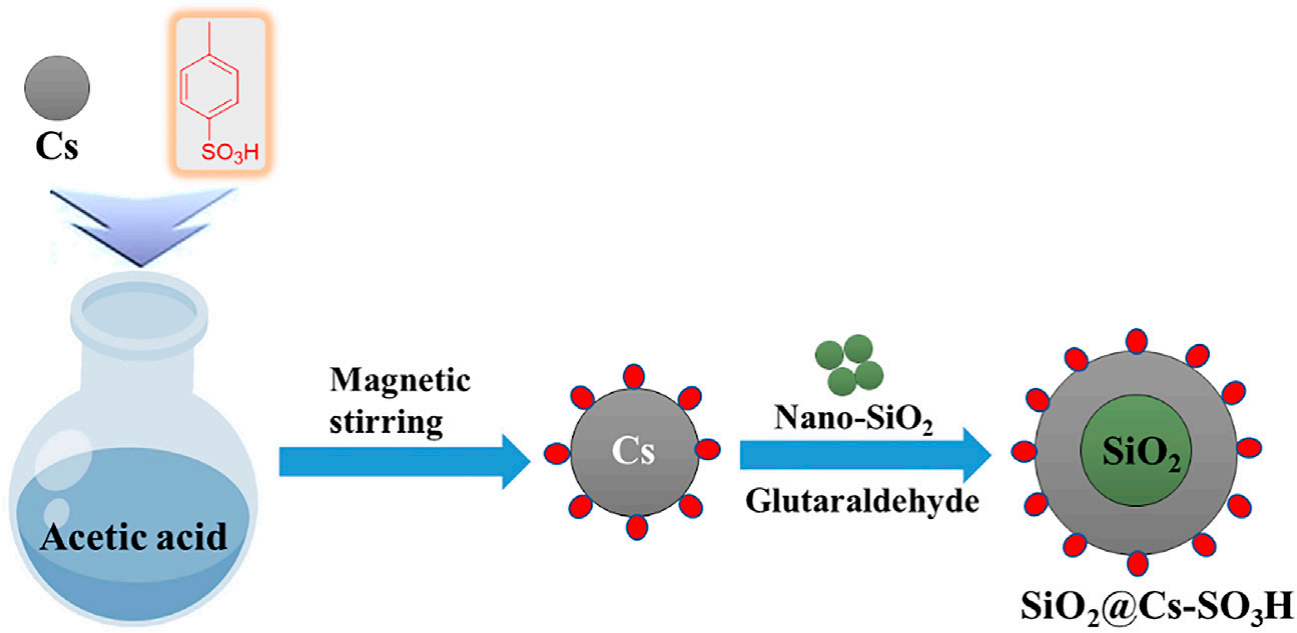
Schematic illustration for the synthesis of SiO_2_@Cs-SO_3_H.

The synthesis diagram of biomass-based solid acid catalyst SiO_2_@Cs-SO_3_H is shown in Scheme 1.

#### 2.2.1 Sulfonation of Chitosan

0.5 g chitosan was added to 50 ml 1 wt% acetic acid solution and stirred magnetically for 10 min at room temperature. Then, 5.7 g *p*-toluenesulfonic acid monohydrate was added to the chitosan acetic acid solution and strongly stirred until the solid was dissolved.

#### 2.2.2 Preparation of SiO_2_ @Cs-SO_3_H Catalyst

0.1 g SiO_2_ and 1 ml glutaraldehyde were added to the sulfonated chitosan solution followed by stirring at room temperature for 2 h. The resulting solution was then dried overnight at 80°C, and the as-synthesized solid materials were ground and repeatedly washed with anhydrous ethanol until the filtrate became pH = 7. Finally, the so-called catalyst SiO_2_@Cs-SO_3_H was obtained after being dried at 80°C for 4 h.

### 2.3 Catalyst Characterization

X-ray diffraction (XRD) was measured by Tongda TD-3500 X-ray diffractometer (Cu K*α* radiation) to characterize the crystallinity and structure of the catalyst. The functional groups of the catalyst were determined by KBr compression using Nicolet 360 FT-IR (Fourier-transform infrared apparatus). The thermal stability of the catalyst in the nitrogen atmosphere was detected by PerkinElmer TGA 47 thermogravimetric analyzer. The ammonia temperature-programmed desorption (NH_3_-TPD) measurements were performed on the AutoChem 2920 chemisorption apparatus with a thermal conductivity detector (TCD) to detect the acidity of the catalyst. The Bruner-Emmett-Teller (BET) method and N_2_ adsorption-desorption apparatus (ASAP 2460, McMerric Instruments Co., Ltd.) were used to check the physical properties of the catalytic material (specific surface area, pore-volume, and pore diameter, respectively).

### 2.4 Esterification of Oleic Acid With Methanol

The catalytic performance of SiO_2_@Cs-SO_3_H was determined by the esterification of oleic acid and methanol for biodiesel synthesis. To be specific, a mixture of 1 g oleic acid (acid value: 200 mg KOH/g), 4.3 ml methanol, and 0.06 g catalyst were added into a 15 ml thick wall pressure tube (Beijing Synthware Glass Instrument Co. Ltd.), and the encapsulated tube was allowed to proceed in an oil bath at 650 rpm using different temperatures for a specific time. After the reaction, petroleum ether was added to extract the reaction products. Finally, the petroleum ether was removed by vacuum distillation. The acid value (AV) of the product was detected by 0.1 M KOH solution titration using phenolphthalein as an indicator (Lin et al., 2021). The yield of biodiesel was determined according to the change of AV before and after oleic acid reaction, and the calculation formula was as follows:

$$\begin{array}{l} Biodiesel\;Yield\left(\%\right)=\frac{AV_{0}-AV_{1}}{AV_{0}}\times \mathrm{100}\%\\ AV_{1}=\frac{56.1\times 0.1\times V_{KOH}}{m} \end{array}$$ (1)

Where AV_0_ and AV_1_ are the acid value of oleic acid and products, respectively; The numbers 56.1 and 0.1 in Eq. 1 are the relative molecular mass and solution concentration (0.1 mol/L) of KOH, respectively; V_*KOH*_ is the volume of KOH titrated; m is the mass of the product.

### 2.5 Response Surface Methodology (RSM)

The factors affecting the esterification of oleic acid with methanol were optimized by the Box-Behnken design (BBD) experiment of RSM. In this process, four factors and three levels were adopted to design the experiment, in which the biodiesel yield was regarded as the response value, and the temperature, time, methanol to oleic acid molar ratio, and the catalyst dosage were served as the main four experimental parameters. Accordingly, a total of 29 experiments were designed in a random order manner. The detailed symbols, names of each parameter, and their ranges and levels are shown in Table 1. Moreover, the relationship between response values and parameters was studied by the least square multiple regression method. The second-order polynomial was fitted to establish the model (Kumar and Singh, 2019; Hossain et al., 2021).

$$\begin{array}{l} \text{Y}=\beta_{\text{0}}\;+\;\beta_{1}\text{A }+\;\beta_{2}\text{B }+\;\beta_{3}\text{C }+\;\beta_{4}\text{D }+\;\beta_{5}\text{AB }+\;\beta_{6}\text{AC }+\;\beta_{7}\text{AD }+\;\\ \beta_{8}\text{BC }+\;\beta_{9}\text{BD }+\;\beta_{10}\text{CD }+\;\beta_{11}{\text{A}}^{2}\;+\;\beta_{12}{\text{B}}^{2}\;+\;\beta_{13}{\text{C}}^{2}\;+\;\beta_{14}{\text{D}}^{2}\;+\;\varepsilon \end{array}$$ (2)

Where Y represents the response prediction value, *β*_*0*_ refers the intercept term, *β*_*1*_ to *β*_*14*_ are the linear effect coefficient, the quadratic coefficient of the crossover and square effect, and *ε* implies the error.

**TABLE 1:** Experimental parameters and range of response surface methodology optimization.

Independent variables	Symbols	Range and levels		
		−1	0	1
Temperature	A	80	90	100
Time	B	3	4	5
Catalyst dosage	C	4	6	8
Methanol to oleic acid molar ratio	D	25	30	35

Analysis of variance (ANOVA) was used to perform statistical analysis on the model, and the influence of independent experimental variables along with their interactions on the response value (biodiesel yield) was also investigated. The *p*-value plays a decisive role in the probability error of the model and the significance of each variable. When the *p*-value is below 0.05%, it indicates that the variable has statistical significance. In addition, a response surface diagram was drawn to demonstrate the influence of the interaction between univariate and bivariate on the yield of biodiesel, and the optimal value was also determined accordingly.

### 2.6 Reusability

The reusability of SiO_2_@Cs-SO_3_H was evaluated under the optimal experimental conditions. After the reaction was finished, the catalyst was separated by centrifugation followed by washing with anhydrous ethanol 3 times to remove the impurities attached to the catalyst. After washing, the catalyst was dried in a vacuum drying oven at 80°C for 8 h to remove the solvent. Then, the as-dried recycled catalyst was employed for the next optimization experiment. The recycling process of the catalyst was repeated for five consecutive runs.

## 3 Results and Discussion

### 3.1 Catalyst Characterization

**FIGURE 1:**
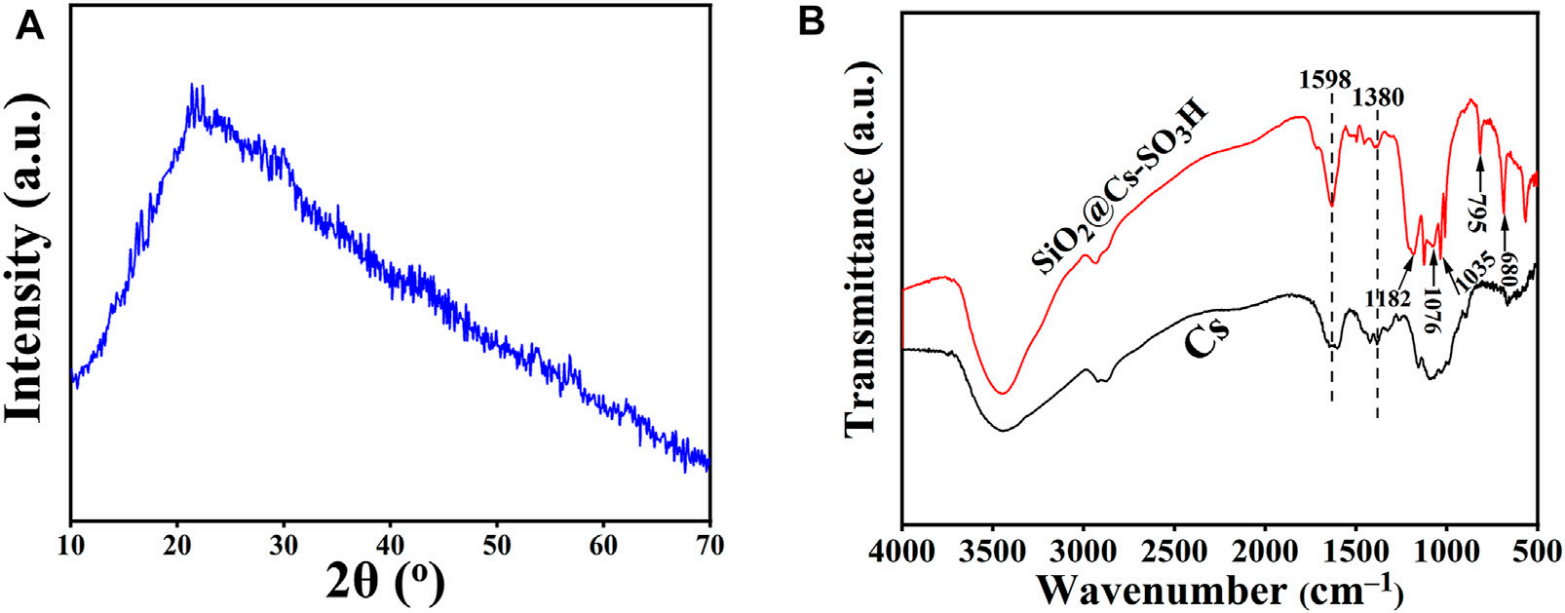
**(A)** XRD spectrum of SiO_2_@Cs-SO_3_H, **(B)** FI-IR spectra of Cs and SiO_2_@Cs-SO_3_H

The structure and crystallinity SiO_2_@Cs-SO_3_H samples were characterized by XRD. As shown in Figure 1A, SiO_2_@Cs-SO_3_H samples showed a broad diffraction peak within the range of 2*θ* = 10°–30°, corresponding to the characteristic peaks of Cs (Ranjani et al., 2019; Sabar et al., 2020). However, the absorption peak of SiO_2_ was not highlighted in the figure, which may be due to the amorphous state of nano-SiO_2_ (Thangaraj et al., 2019). With respect to this, the existence of SiO_2_ was further proved by FT-IR spectroscopy. Figure 1B shows the FT-IR spectra of SiO_2_@Cs-SO_3_H and Cs. The absorption peaks observed in both the two samples at 1,380 cm^−1^ and 1,598 cm^−1^ are attributed to the C-O and N-H functional groups, respectively, and these should be typical characteristic peaks of Cs (Saengprachum et al., 2019). The obvious peaks of SiO_2_@Cs-SO_3_H at 1,182 cm^−1^ and 1,035 cm^−1^ are derived from the stretching vibration of O=S=O, indicating the successful functionalization of the active sulfonic acid group on the Cs (Nata et al., 2017). Meanwhile, the absorption peaks shown at 1,076 cm^−1^, 795 cm^−1^, and 680 cm^−1^ correspond to the anti-symmetrical stretching vibration, symmetrical stretching, and bending of Si-O-Si, respectively (Owoeye et al., 2020). These indicate that the sulfonated chitosan has been successfully coated onto SiO_2_.

**FIGURE 2:**
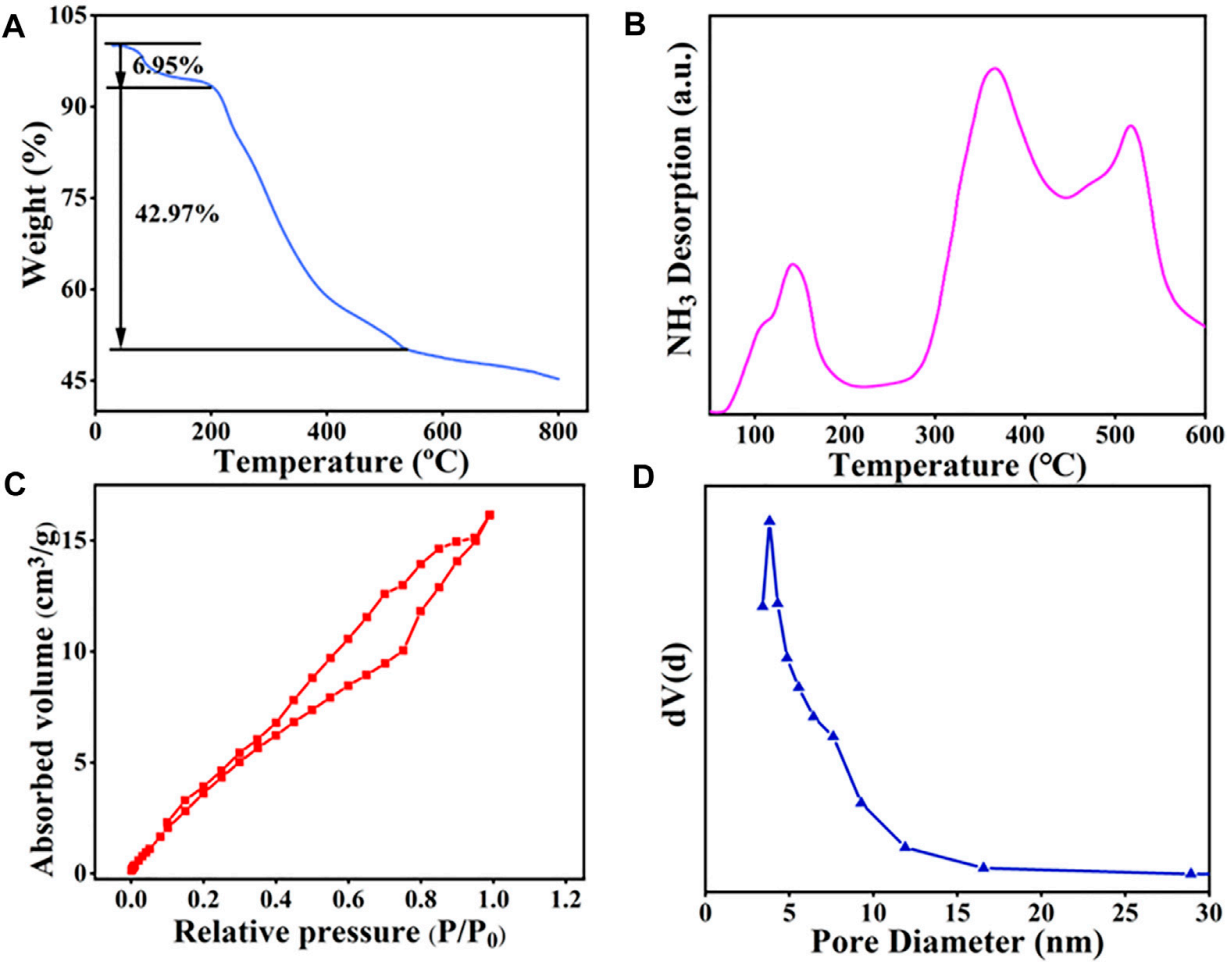
SiO_2_@Cs-SO_3_H catalyst diagram of **(A)** TGA, **(B)** NH_3_-TPD, **(C)** BET, and **(D)** pore size distribution.

The thermal stability of the prepared catalyst was studied by TGA analysis. From Figure 2A, it can be observed that the weight loss of SiO_2_@Cs-SO_3_H mainly occurs in two stages. When the temperature is below 205°C, the weight loss of the catalyst determined as 6.95 wt% should be ascribed to the loss of water adsorbed on the catalyst. The second peak existing in the range of 205–540°C (weight loss of 42.97 wt%) was related to the polymeric network decomposition and the formation of carbon materials. However, the mass of the catalyst did not decrease significantly after 540°C, indicating that the carbon material produced gradually became stable. In short, the catalyst showed good thermal stability and it can completely meet the demand of catalytic esterification reaction to produce biodiesel.

NH_3_-TPD was used to investigate the relative acidity density and acidic strength of SiO_2_@Cs-SO_3_H. As depicted in Figure 2B, the peaks shown in the profile were derived from the desorption of NH_3_ at acidic sites on the sample, and the desorption temperature was related to the acid strength of SiO_2_@Cs-SO_3_H. As illustrated in Figure 2B, the NH_3_-TPD profile shows three peaks within the range of 50–600°C. The peaks in the temperature ranges of 110–200°C, 310–400°C, and 450–520°C correspond to the sites of a weak acid (-OH), medium strong acid (-SO_3_H) and strong acid (-SO_3_H), respectively. Meanwhile, the measurement result showed that SiO_2_@Cs-SO_3_H possessed a higher acid density of 3.47 mmol/g, which were superior to the reported sulfonated solid acid catalysts such as SAC (1.50 mmol/g) (Lathiya et al., 2018), ASHC-SO_3_H (1.40 mmol/g) (Cao et al., 2021), and PMB-SO_3_H (1.92 mmol/g) (Quah et al., 2020). The specific surface area, pore size, and pore volume of the SiO_2_@Cs-SO_3_H catalyst were measured by BET-BJH, as shown in Figures 2C,D. According to the results, the catalyst exhibited a specific surface area of 21.82 m^2^/g, a pore volume of 0.022 cm^2^/g, and a pore size distribution between 2 and 20 nm. Compared with the sulfonated chitosan catalysts that were reported in the previous literature (Wang et al., 2019; Zhang and Sun, 2020), SiO_2_@Cs-SO_3_H demonstrated a benign porous structure, implying that SiO_2_@Cs-SO_3_H will be conducive to esterification for biodiesel synthesis. It is worth mentioning that when nano-SiO_2_ with good surface efficiency and large surface area was used as the suitable carrier, of crucial significance was to improve the specific surface area of the catalyst, thus leading to better catalytic performance accordingly.

### 3.2 Esterification of Oleic Acid to Biodiesel

The prepared SiO_2_@Cs-SO_3_H catalyst from sulfonated chitosan was further applied to catalyze the esterification reaction of oleic acid with methanol for the production of biodiesel. The effects of reaction temperature (70–110°C), reaction time (1–5 h), catalyst dosage (0–8 wt%), and the methanol to OA molar ratio (15/1–35/1) on biodiesel yield were studied by single-factor experiments.

#### 3.2.1 Reaction Temperature

**FIGURE 3:**
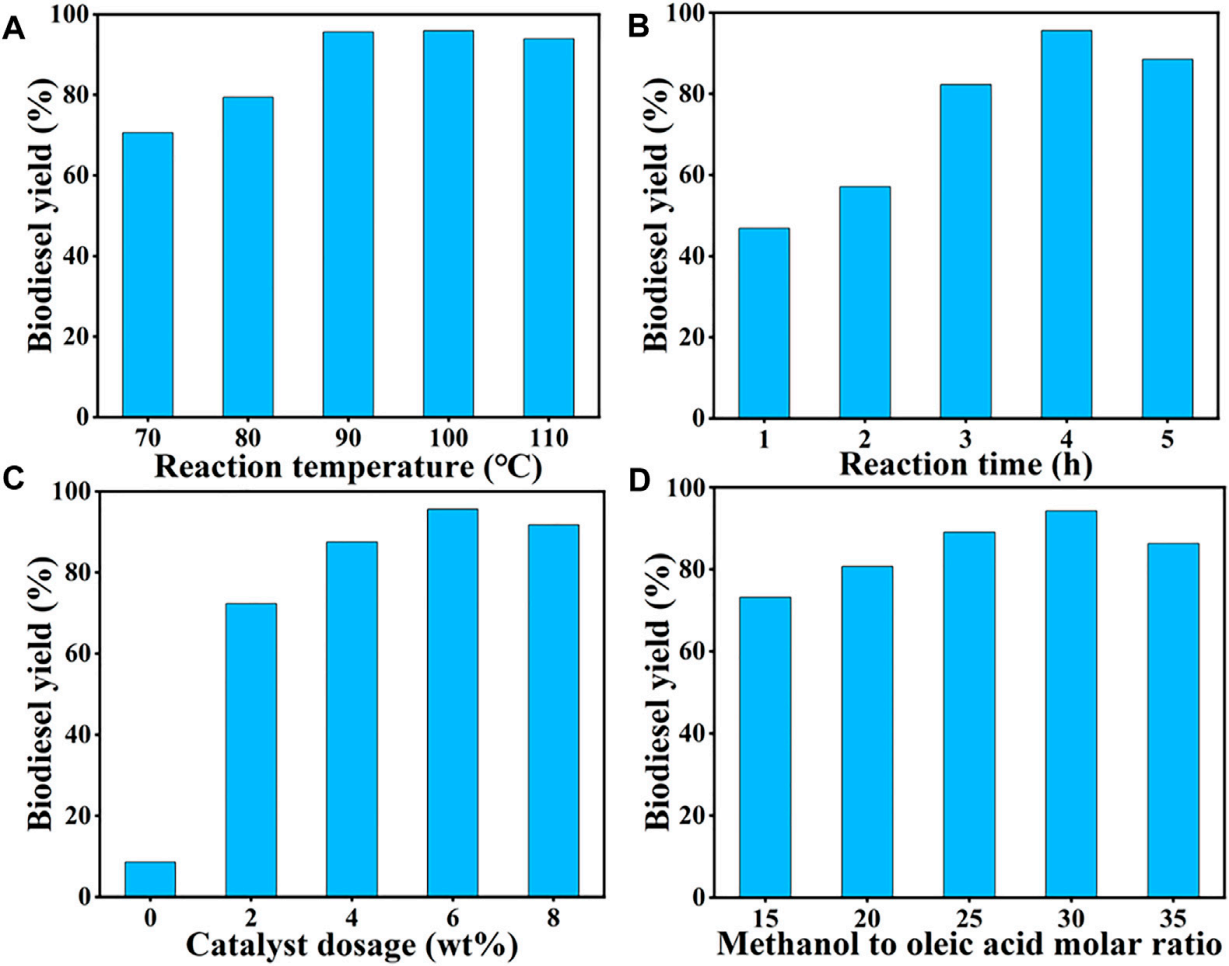
Single-factor optimization of SiO_2_@Cs-SO_3_H catalyst for oleic acid esterification reaction: **(A)** reaction temperature, **(B)** reaction time, **(C)** catalyst dosage, and **(D)** methanol to oleic acid molar ratio.

Generally, the reaction temperature is a significant variable affecting the reaction rate in the esterification of OA with methanol (Zhang et al., 2018). As shown in Figure 3A, the influence of temperature on the yield of biodiesel was investigated in the temperature range of 70–110°C. It was found that with the temperature rising from 70 to 90°C, the biodiesel yield gradually increased to more than 90%. Subsequently, as the temperature continued to rise, the yield of biodiesel decreased slightly, which may be due to the decrease of methanol feedstock caused by the higher temperature. Therefore, 90°C was selected as the appropriate reaction temperature.

#### 3.2.2 Reaction Time

The reaction time also has a certain influence on biodiesel yield. From the point of kinetic, the esterification of OA with methanol requires a certain amount of time for mass transfer to reach an equilibrium state (Pan et al., 2017). Figure 3B shows the effect of reaction time on biodiesel yield at 90°C, methanol to OA molar ratio of 30/1, and catalyst dosage of 6 wt%, and the highest yield of 96% was obtained after 4 h of reaction. Thus, 4 h was determined as the optimal reaction time.

#### 3.2.3 Catalyst Dosage

The amount of catalyst is also an important parameter to evaluate its catalytic capacity for OA esterification reaction, wherein the number of active sites is closely related to the yield of biodiesel (Wang et al., 2021). As shown in Figure 3C, only 8.57% of the biodiesel yield was obtained without any catalyst. With the increase of catalyst amount from 0 wt% to 6 wt%, the corresponding biodiesel yield reached over 90%. However, when the catalyst amount exceeded 6 wt%, the biodiesel yield showed a downward trend. This could be explained that since the increase of the catalyst dosage proceeded, the number of catalytic active sites also raised, thus promoting the esterification reaction towards the biodiesel synthesis. It should be noted that too much catalyst would hinder mass transfer between the catalyst and the reactants, slowing down the reaction rate ultimately. Therefore, 6 wt% was selected as the suitable dosage of catalyst.

#### 3.2.4 Methanol to OA Molar Ratio

Apart from the above three factors, the amount of methanol also played a crucial role in the esterification of OA. It is certain that appropriately increasing the amount of alcohol can promote the forward reaction, thus improving the yield of biodiesel accordingly (Xie and Wang, 2021). As shown in Figure 3D, the influence of alcohol dosage on biodiesel yield was studied with different molar ratios (15/1–35/1), while remaining the other variables as the same. It was determined that the biodiesel yield showed an upward trend with the increase of molar ratio, of which the highest yield of 95% was observed at 30/1. However, excessive methanol ultra-dilution the concentration of the reaction system and reduces the chance of OA arriving at the catalytic active site, thus reducing the yield of biodiesel. Thus, 30/1 was determined as the appropriate molar ratio.

### 3.3 Optimization of Biodiesel Production by RSM

To further optimize the reaction conditions of the SiO_2_@Cs-SO_3_H catalyzing oleic acid esterification reaction to obtain the best biodiesel yield, we further employed the response surface method to randomly design 29 sets of experiments on the basis of the single-factor experimental results in the previous section. The obtained results and the corresponding predicted values of the above Eq. 2 are shown in Table 2. The specific second-order polynomial equation of the design model based on the predicted response value of the four parameters is depicted in Eq. 3.

$$\begin{array}{l} \text{Y}=\;97\text{.}33\;+\;1\text{.}45\text{A }+\text{ 0}\text{.}68\text{B }+\;2\text{.}15\text{C }+\text{ 0}\text{.}78\text{D }-\;1\text{.}09\text{AB }-\text{ 0}\text{.}18\text{AC }-\text{ 0}\text{.}02\text{AD }\\ +\text{ 0}\text{.}12\text{BC }+\text{ 0}\text{.}86\text{BD }+\;1\text{.}09\text{CD }-\;1{\text{.}09\text{A}}^{2}\;-\text{ 0}{\text{.}42\text{B}}^{2}\;-\;1{\text{.}66\text{C}}^{2}\;-\;1{\text{.}15\text{D}}^{2} \end{array}$$ (3)

**TABLE 2:** Experimental design and results of oleic acid esterification reaction catalyzed by SiO_2_@Cs-SO_3_H.

Run	A: Temperature (°C)	B: Time (h)	C: Catalyst dosage (wt%)	D: Methanol to oleic acid molar ratio	Experimental biodiesel yield (%)	Predicted biodiesel yield (%)
1	90	3	4	30	92.64	92.53
2	90	4	6	30	97.33	97.33
3	80	4	6	35	94.77	94.43
4	100	3	6	30	97.34	97.67
5	90	4	4	25	92.96	92.68
6	80	4	4	30	90.24	90.17
7	80	5	6	30	96.44	96.13
8	100	5	6	30	97.17	96.87
9	80	4	6	25	92.66	92.84
10	90	4	8	25	95.23	94.8
11	90	5	6	35	97.98	98.07
12	100	4	6	35	97.54	97.3
13	90	4	6	30	96.34	97.33
14	80	3	6	30	92.27	92.6
15	90	5	4	30	93.49	93.66
16	90	4	4	35	91.59	92.05
17	90	5	6	25	94.50	94.81
18	100	4	8	30	97.27	97.38
19	90	5	8	30	98.15	98.2
20	90	4	6	30	97.82	97.33
21	90	3	6	35	95.26	94.99
22	100	4	4	30	94.85	94.68
23	100	4	6	25	95.51	95.78
24	90	3	6	25	95.20	95.15
25	90	4	6	30	97.54	97.33
26	90	4	8	35	98.14	97.54
27	90	4	6	30	97.62	97.33
28	90	3	8	30	96.82	96.59
29	80	4	8	30	95.88	96.08

**FIGURE 4:**
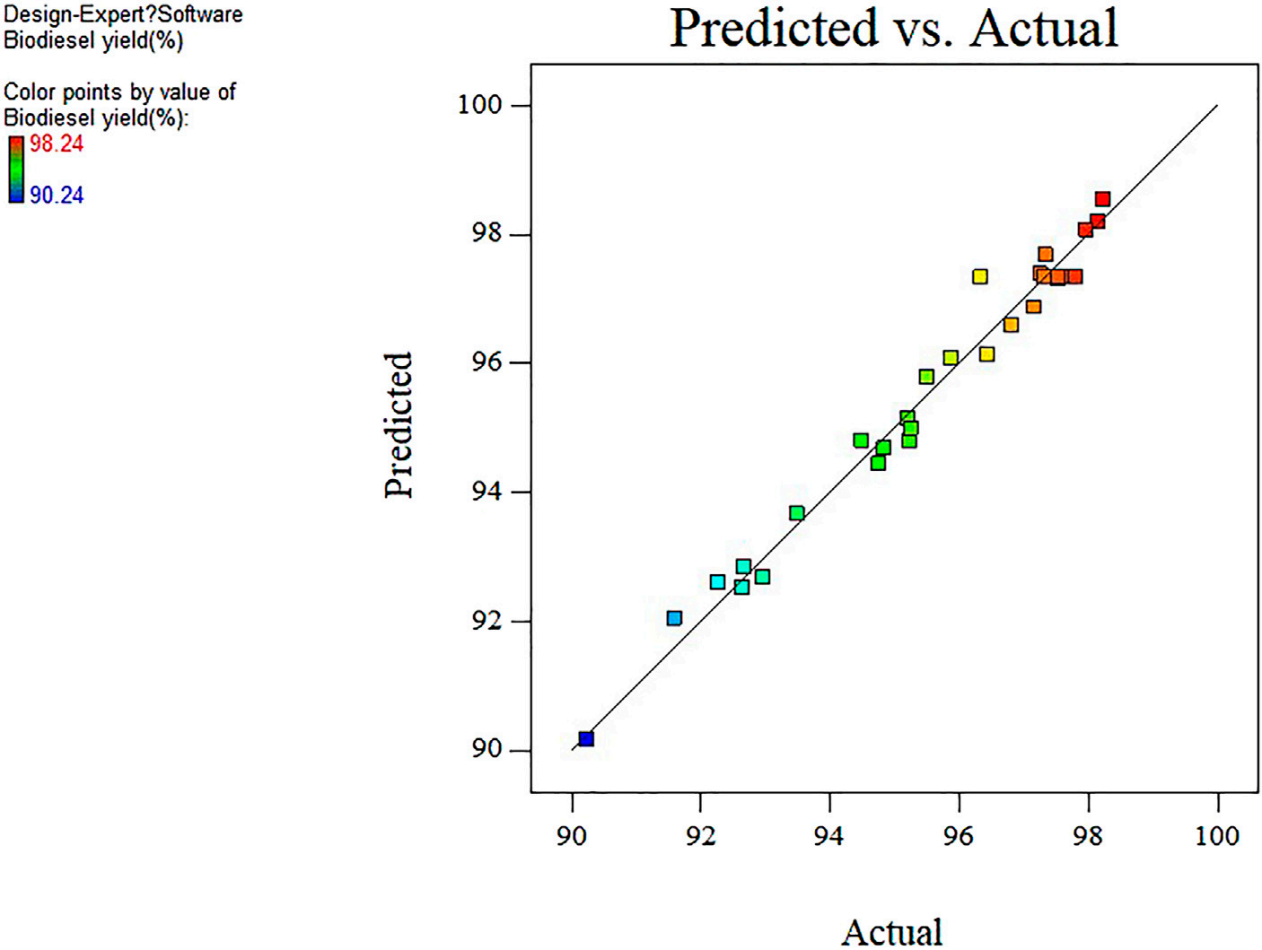
Comparison of experimental and predicted values.

Analysis of variance was performed on the experimental design model as exhibited in Table 2, and the corresponding results are presented in Table 3. The F and *p*-value represent the significance of the entire model and the model term respectively. The larger the F value, the more significant the model and the better the fit. The smaller the *p*-value, the more important the model term is for the response value. In general, the *p*-value < 0.05 is highly significant of the model variables (Sulaiman et al., 2017). According to the results in Table 3, it can be observed that the reaction parameters including A, B, C, and D can be determined to be highly significant. Meanwhile, for the high F value (45.57) and low *p*-value (<0.0001) of the model, it can be seen that the selected quadratic polynomial regression model to optimize the yield of biodiesel is significant. The “Lack of Fit F-value” of 0.47 indicates that lack of fit is not significant compared with the pure error, showing that the model fits the experimental data sufficiently. Furthermore, the coefficient of determination *R* ^2^ of 97.85% indicates that the obtained model can explain 97.85% of the response variability, indicating that the model has high reliability. The adjusted *R* ^2^ of 95.71% and the predicted *R* ^2^ of 91.76% are considered to be reasonably consistent because their difference is less than 0.2. Thus, it can be concluded that the fit of the model is more significant. To explain the reliability and applicability of the model, the experimental real value is compared with the predicted value (Figure 4). There is a low deviation between the predicted value and the real value. Therefore, these have proven that the quadratic polynomial regression model can match the data well, and the system response is generated within the scope of independent variable research.

**TABLE 3:** ANOVA on the RSM experimental design data.

Source	Sum of squares	Df	Mean square	F value	*p*-value prob > F	
Model	134.32	14	9.59	45.57	<0.0001	significant
A: Temperature	25.29	1	25.29	120.11	<0.0001	
B: Time	5.6	1	5.6	26.61	0.0001	
C: Catalyst dosage	55.56	1	55.56	263.88	<0.0001	
D: Methanol/OA molar ratio	7.24	1	7.24	34.38	<0.0001	
AB	4.71	1	4.71	22.37	0.0003	
AC	2.59	1	2.59	12.31	0.0035	
AD	0.0016	1	0.0016	0.0076	0.9318	
BC	0.058	1	0.058	0.27	0.6091	
BD	2.92	1	2.92	13.89	0.0023	
CD	4.8	1	4.8	22.78	0.0003	
A^2^	7.69	1	7.69	36.55	<0.0001	
B^2^	1.17	1	1.17	5.54	0.0337	
C^2^	17.91	1	17.91	85.07	<0.0001	
D^2^	8.6	1	8.6	40.86	<0.0001	
Residual	2.95	14	0.21			
Lack of Fit	1.6	10	0.16	0.47	0.8455	not significant
Pure Error	1.35	4	0.34			
Cor Total	137.27	28				

**FIGURE 5:**
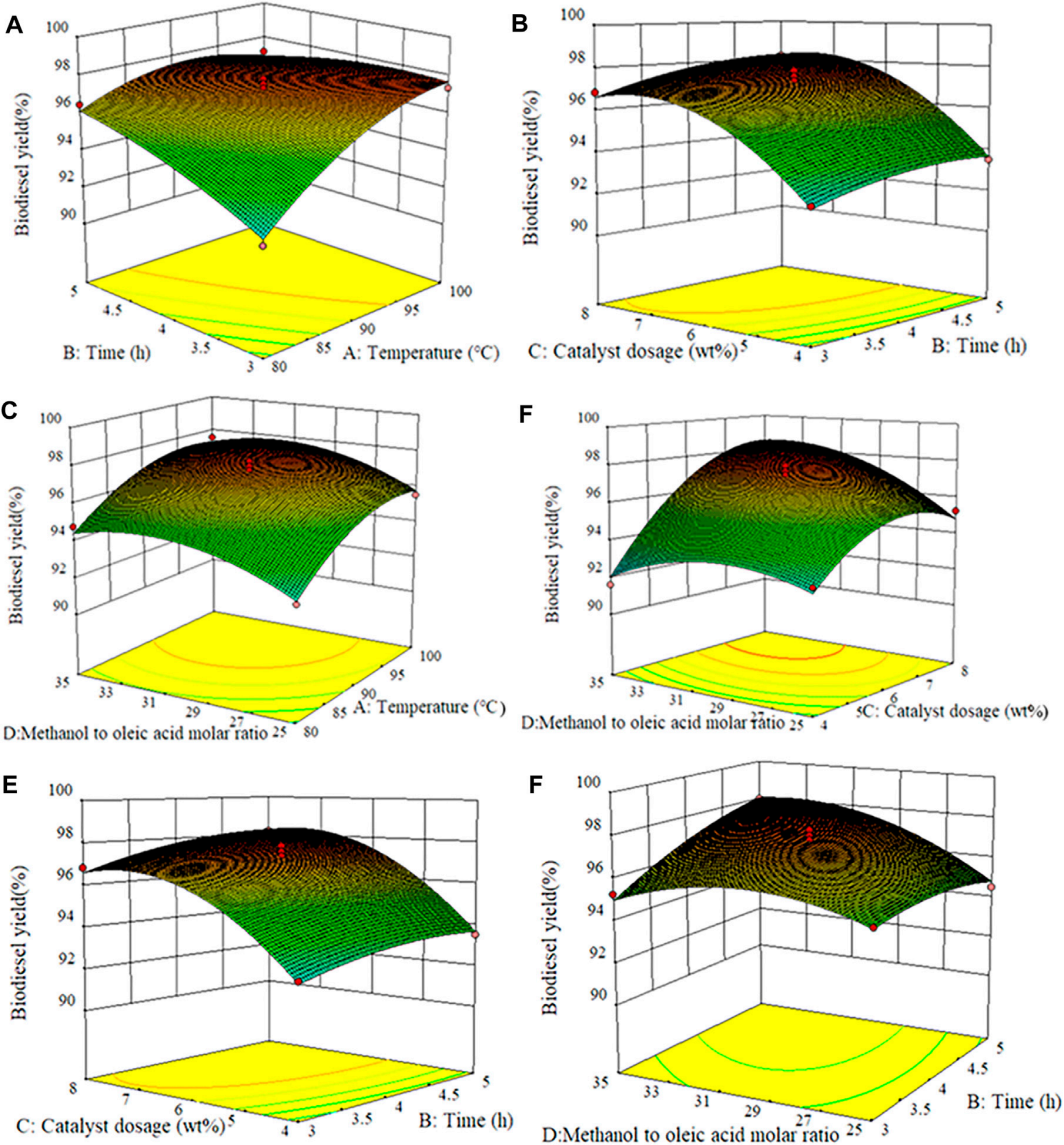
Surface response plots of different experimental variables against biodiesel yield. **(A)** Temperature and time, **(B)** time and catalyst dosage, **(C)** methanol to oleic acid molar ratio and temperature, **(D)** catalyst dosage and methanol to oleic acid molar ratio, **(E)** catalyst dosage and time, and **(F)** methanol to oleic acid molar ratio and time.

Through the three-dimensional surface graph, the influence of the interaction of any two independent variables on biodiesel yield was studied, while the coding values of the other two parameters were kept at zero, as shown in Figure 5. Figure 5A displays the interaction effect of time and temperature, and catalyst dosage was 6 wt% and methanol to oleic acid molar ratio was 30:1. The extension of reaction time shows no significant impact on the yield of biodiesel in the lower reaction temperature range, which may be that the reaction requires a higher temperature to improve the reaction rate (Wang et al., 2018). However, with the increase of the temperature, it can be found that higher biodiesel yield can be reached in a relatively short time, indicating that the influence of reaction temperature on biodiesel yield was more significant than the reaction time. This can also be observed from the higher F value (120.11) of the reaction temperature. Figure 5B and Figure 5C show the interaction of temperature with catalyst dosage and molar ratio, respectively. For low temperature and catalyst dosage, the biodiesel yield was low. However, when the amount of catalyst and temperature increased, the biodiesel yield gradually moved to the high-value region, and the effect of the catalyst dosage on the yield of biodiesel was greater than that of the reaction temperature. This is attributed to the addition of catalytic active sites that can promote the reaction more effectively and quickly, but a superfluous amount will inversely hinder the mass transfer between substances, slowing down the reaction rate ultimately (Cao et al., 2021). Similarly, Figure 5C shows that the biodiesel yield increases with the increase of reaction temperature and the molar ratio of methanol to oleic acid, but when the molar ratio increases to a certain extent, the biodiesel yield takes on a downtrend. This was because the increase of methanol dosage is conducive to the positive occurrence of the reaction, thus resulting in the improvement of product yield. However, excessive methanol will dilute the concentration of the reaction system, and the inverse reaction rate will be increased with the increase of the number of product esters, both of which are unfavorable for the formation of products (Lim et al., 2020). In addition, compared with the molar ratio, the influence of temperature on the yield of biodiesel was more significant.

The conjugate effect of the catalyst amount and methanol/OA molar ratio on biodiesel yield was depicted in Figure 5D. The reaction temperature and time were kept at 90°C and 4 h, respectively. It can be seen from Figure 5D that the yield of biodiesel was constantly increased with the molar ratio of methanol to oleic acid and catalyst dosage increased. In the esterification reaction, the low molar ratio of methanol to oleic acid is adopted, the increase of catalyst dosage demonstrated a weak effect on the improvement of biodiesel yield. This should be due to the fact that the reversibility of the esterification reaction usually requires a higher methanol/OA molar ratio. However, an excessive amount of methanol will dilute the concentration of catalyst in the whole system, resulting in a decrease in the concentration of active center per unit volume (Nongbe et al., 2017). Figure 5E and Figure 5F show the interaction of time with the catalyst amount and the molar ratio of methanol to OA, respectively. As mentioned earlier, the biodiesel yield continues to increase with the increment of time, the amount of catalyst, and the molar ratio.

The experimental parameters were optimized according to the RSM to obtain the best biodiesel yield. The optimum conditions were obtained as follows: reaction temperature of 92°C, reaction time of 4.1 h, catalyst dosage of 6.8 wt%, and methanol to oleic acid molar ratio of 31.4/1. Correspondingly, the predicted biodiesel yield was as high as 98.4%. For the effectiveness of the model, the corresponding experiments were conducted under the predicted conditions, and the experimental biodiesel yield of 98.2% was very close to the predicted value, verifying that the model had certain reliability and effectiveness in predicting the biodiesel yield in this study.

### 3.4 Reusability of Catalyst

**FIGURE 6:**
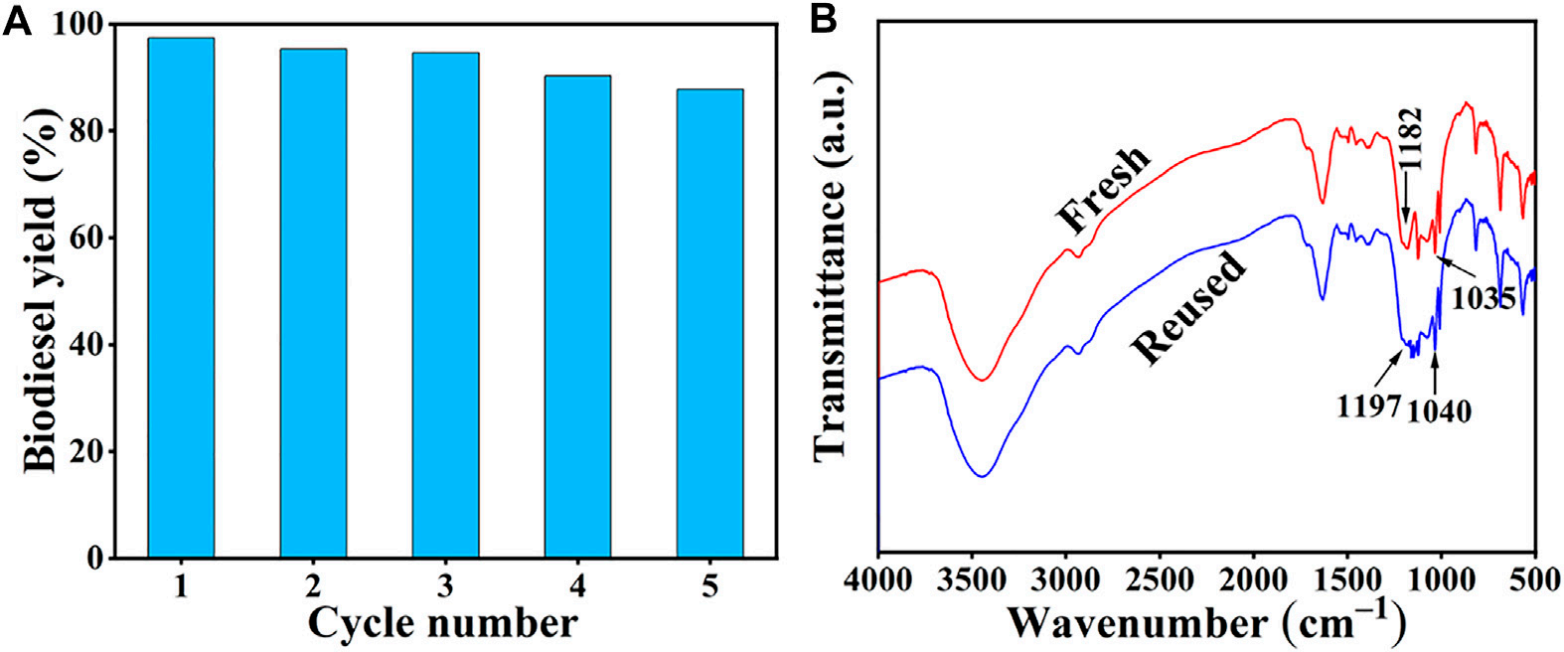
**(A)** Reusability of SiO_2_@Cs-SO_3_H catalyst, **(B)** FI-IR spectra before and after repeated use of SiO_2_@Cs-SO_3_H (Methanol to oleic acid molar ratio of 31.4/1, 6.8 wt% and 92°C for 4.1 h).

Reusability is considered to be an important guideline to assess the performance of the catalyst, and efficient catalyst recycle can also reduce the biodiesel cost, to some extent (Dai et al., 2021). Therefore, we conducted five consecutive reactions under the optimized experimental conditions to determine the reusability of the SiO_2_@Cs-SO_3_H catalyst in oleic acid esterification reaction. The specific steps are as follows: after each reaction, the catalyst obtained through centrifugation was washed 3 times with anhydrous ethanol to remove the impurities attached to the catalyst, and then dried at 80°C to remove the solvent. The dried catalyst was used for the next experiment. The tested results of SiO_2_@Cs-SO_3_H catalyst after five recycles are shown in Figure 6A, wherein 87.0% biodiesel yield for the fifth recycle can still be reached, showing the relatively good reusability of SiO_2_@Cs-SO_3_H. More importantly, Figure 6B shows the infrared spectra of the catalysts before and after use, whereas the important absorption peak of the sulfonic acid -SO_3_H group still exists in the reused catalyst. Nonetheless, the intensity of the absorption peak is relatively weak compared with the fresh catalyst, indicating that part of -SO_3_H was lost in the recycling process, which may be the reason for the decrease of biodiesel yield.

## 4 Conclusion

Sulfonated chitosan-derived SiO_2_@Cs-SO_3_H solid acid catalyst was synthesized by an easy method at room temperature. The SiO_2_@Cs-SO_3_H catalyst exhibited a high acidity (3.47 mmol/g) and large specific surface area (21.82 m^2^/g), showing high catalytic activity for the esterification of oleic acid and methanol. The reaction temperature, reaction time, catalyst amount, and molar ratio of methanol to oleic acid were optimized and analyzed by RSM to obtain the best biodiesel yield. Accordingly, the ideal reaction conditions were determined: 92°C, 4.1 h, catalyst dosage of 6.8 wt%, and methanol to oleic acid molar ratio of 31.4/1. In this case, the maximum biodiesel yield (98.2%) was obtained. In addition, the catalyst also had good reusability, and the biodiesel yield of 87.0% can still be reached after repeated use of 5 times. In conclusion, the SiO_2_@Cs-SO_3_H solid acid catalyst prepared from Cs biomass had excellent catalytic performance for the esterification of FFAs, which shows a good application prospect in the biodiesel production field.

## Data Availability

The original contributions presented in the study are included in the article/Supplementary Material, further inquiries can be directed to the corresponding author.
